# Patterns and predictors of help-seeking contacts with health services and general practitioner detection of suicidality prior to suicide: a cohort analysis of suicides occurring over a two-year period

**DOI:** 10.1186/s12888-016-0824-7

**Published:** 2016-04-30

**Authors:** Gerard Leavey, Michael Rosato, Karen Galway, Lynette Hughes, Sharon Mallon, Janeet Rondon

**Affiliations:** Bamford Centre for Mental Health & Wellbeing, Ulster University, Cromore Road, Coleraine, BT52 1SA Londonderry Northern Ireland; School of Nursing and Midwifery, Queen’s University Belfast, Medical Biology Building, 97 Lisburn Road, Belfast, BT 9 7BL Northern Ireland; Faculty of Health and Social Care, Horlock Building, Open University, Milton Keynes, MK7 6AA Northern Ireland

**Keywords:** Suicide predictors, Suicidality, Help-seeking, Health services, General practice

## Abstract

**Background:**

Contact with primary care and psychiatric services prior to suicide may be considerable, presenting opportunities for intervention. However, there is scant knowledge on the frequency, nature and determinants of contact.

**Method:**

Retrospective cohort study-an analysis of deaths recorded as suicide by the Northern Ireland Coroner’s Office linked with data from General Practice patient records over a 2 year period

**Results:**

Eighty-seven per cent of suicides were in contact with General Practice services in the 12 months before suicide. The frequency of contact with services was considerable, particularly among patients with a common mental disorder or substance misuse problems. A diagnosis of psychiatric problems was absent in 40 % of suicides. Excluding suicide attempts, the main predictors of a noted general practitioner concern for patient suicidality are male gender, frequency of consultations, diagnosis of mental illness and substance misuse.

**Conclusions:**

Despite widespread and frequent contact, a substantial proportion of suicidal people were undiagnosed and untreated for mental health problems. General Practitioner alertness to suicidality may be too narrowly focused.

## Background

In Western societies the main factors associated with suicide include mental illness, gender, age, social class, unemployment, poor physical health and social isolation [1, 2]. These factors are also related to help-seeking for mental health problems. Contact with primary care services prior to suicide is common, providing an opportunity for intervention for people recognised as vulnerable. Luoma et al. [3] indicate contact averages of 77 % (range = 55–80 %) within 12-months, and 45 % (20–76 %) within 1-month of death. In England, Pearson et al. [4] found that 91 % of individuals had consulted their general practitioner (GP) at least once in the year before death, with an average of seven consultations, almost double the general population rate [5]. A recent case-control study of suicides in England over a 10-year period showed that suicide risk is associated with increasing frequency of GP consultations, particularly in the three months prior to suicide, with highest risk among patients who consulted with their GP more than twenty-four times in the final year. While approximately one-third of suicides may be in contact with mental health services during the 12-month period, mental illness was frequently unrecognised by GPs [6]. Less than one third of adults with diagnosable mental disorders seek professional help [7, 8].

### Study aims

To examine predictors of contact with health and social care services in the 12-month period prior to suicide and GP recognition of suicidality.

#### Study design

A retrospective cohort analysis of all deaths recorded as suicide over a 2-year period from 1st March 2007–28th February 2009. The data was obtained from the records of the Coroner Service for Northern Ireland (CSNI) and linked to individual General Practice (GP) patient notes.

### Ethical approval

Permission to access the required data from CSNI and GP records was granted using the ‘research exemption’ Section 33 of the Data Protection Act. Ethical approval for the study was obtained from The UK Office for Research Ethics Committees Northern Ireland (ORECNI).

## Methods

A retrospective cohort study providing an analysis of deaths recorded as suicide by the Northern Ireland Coroner’s Office linked with data from General Practice patient records over a 2 year period.

### Data collection

We gathered data from the CSNI records using a self-designed and piloted pro-forma. We then examined linked patient GP records from the General Records Office. Data extracted for each case included the following: demographic characteristics (e.g., age, gender, marital status and family circumstances); diagnosis and history of mental and physical health problems; and details of the suicide event and its circumstances.

Using postcode data each record was assigned to one of an eight-fold classification of settlement type derived by the Northern Ireland Statistics and Research Agency (NISRA, 2006) ranging from urban to rural locales. For analysis this was summarised into four categories: urban (comprising the Belfast Metropolitan and Derry Urban Areas); larger towns; smaller towns; and rural (comprising small villages with populations less than 1,000 and hamlets and open country).

Two indicators reflecting socioeconomic circumstances derived from occupation data from the Coroners records were included: those in non-voluntary employment (yes/no); and social class (grouped as professional, other classifications derived from stated occupation, and others).

Pre-existing mental health problems (MHP) were summarised into three groups: (i) common mental disorders (CMD) included depression, anxiety, phobias, stress, psychological disorders and Posttraumatic stress disorder (PTSD); (ii) severe mental illness (SMI) comprised schizophrenia, psychosis, bipolar disorder and personality disorders; and (iii) alcohol/drugs problems included those with alcohol and/or drug dependency problems. We used the International Classification of Primary Care (ICPC-2) [9] which permits the classification of the patient’s reason for encounter, problems managed and primary or general health care interventions. Finally, from the GP record we derived data on help-seeking behaviour and service over the twelve months before death. Additionally, we noted where individual GPs made reference to an individual’s potential suicidality.

### Data analysis

These data were systematically checked and validated and analysed using Stata version 13. We used simple descriptive statistics and chi-square tests for differences between categorical variables and the different aspects of health-seeking behaviour relating to suicide. We quantified service access at one month, three months and twelve months prior to death, using means (and medians as appropriate, determined by the Shapiro-Wilks test) and Standard Deviation. *T*-test and Anovas were used to examine difference in frequency of contact. We used stepwise logistic regression to examine the relationships between the selected risk factors and the likelihood of access to services: from consultations with a psychiatrist in the twelve months prior to the suicide; and access to general practitioner services at the noted time points.

## Results

Of the 399 eligible cases of suicide recorded by the Coroner’s Office, GP records were unobtainable for 38 individuals (9.5 % of all cases). These records were excluded from the analysis of service access, which therefore was carried out with a base population of 361. We found no significant differences in gender, age, locale, marital status, living alone or employment between available and unavailable GP records.

The crude overall suicide rate for Northern Ireland for the 2-year period was 11.3 per 100,000 population. Highest rates were recorded in larger towns (14.1/100,000). Table 1 shows the distribution of suicides, by gender and the main parameters known to influence suicide: 80 % of suicides were by males; age at suicide ranged from 11 to 83 years (mean 39 years and SD = 16.3) - with the peak age for males being younger than for females (25–34 against 35–44 respectively); 31 % of the cohort lived alone at the time of the suicide; 59 % were in paid employment (66 % of males and 31 % of females); and 40 % had recorded a prior suicide attempt (54 % of females and 36 % of males). While 6 % of suicides were to those from a professional social class, and 41 % in a social class definable by occupation - 45 and 24 % to males and females respectively – the majority of suicides (54 %) were to those in a social class comprising a combination of categories (including the retired) derived on a basis other than occupation.

**Table 1:** Suicides in NI (2007–2009): by gender and the main parameters known to influence suicide

	Males	Females	Persons
	(*N* = 321)	(*N* = 78)	(*N* = 399)
	% (95 % CI)	% (95 % CI)	% (95 % CI)
Proportion	80.45 (76.25, 84.07)	19.55 (15.93. 23.75)	
Age (mean & standard deviation)^a^	39.16 (16.21)	39.18 (16.57)	39.16 (16.27)
Age group less than 25	20.25 (16.19, 25.03)	21.79 (13.86, 32.56)	20.55 (16.85, 24.82)
25–34	24.30 (19.90, 29.32)	15.38 (8.84, 25.43)	22.56 (18.70, 26.94)
35–44	19.94 (15.90, 24.70)	28.21 (19.18, 39.41)	21.55 (17.78, 25.88)
45–54	18.07 (14.21, 22.69)	20.51 (12.82, 31.16)	18.55 (15.02, 22.68)
55–64	9.66 (6.86, 13.43)	5.13 (1.90, 13.14)	8.77 (6.36, 11.99)
65 plus	7.79 (5.31, 11.29)	8.97 (4.27, 17.90)	8.02 (5.72, 11.13)
Living alone: yes	33.33 (28.36, 38.71)	21.79 (13.86, 32.56)	31.08 (26.71, 35.81)
In paid work: yes	66.04 (60.66, 71.04)	30.77 (21.37, 42.09)	59.16 (54.23, 63.89)
Social class:			
Professional	5.68 (3.60, 8.85)	6.41 (2.64, 14.75)	5.82 (3.89, 8.62)
Intermediate; semi-routine; routine	44.79 (39.40, 50.34)	24.36 (15.95, 35.33)	40.76 (36.00, 45.70)
Other (including retired)	49.53 (44.02, 55.04)	69.23 (57.91, 78.63)	53.42 (48.46, 58.31)
Locale: Urban	39.88 (34.63, 45.36)	39.74 (29.32, 51.19)	39.85 (35.13, 44.76)
Large towns	32.09 (27.18, 37.42)	33.33 (23.60, 44.73)	32.33 (27.90, 37.10)
Small towns	6.54 (4.29, 17.90)	8.97 (4.27, 17.90)	7.02 (4.88, 9.99)
Rural	21.50 (17.32, 26.36)	17.95 (10.80, 28.32)	20.80 (17.08, 25.09)
Prior mental health diagnosis:			
None	43.86 (38.17, 49.71)	30.43 (20.54, 42.54)	41.24 (36.20, 46.47)
Common mental disorders	29.82 (24.77, 35.43)	44.93 (33.40, 57.03)	32.77 (28.06, 35.86)
Severe mental illness	10.53 (7.44, 14.69)	17.39 (10.01, 28.50)	11.86 (8.87, 15.69)
Alcohol/drugs related	15.79 (11.98, 20.53)	7.25 (2.98, 16.58)	14.12 (10.86, 18.18)
Previous attempts (one or more): yes	36.45 (31.34, 41.89)	53.85 (42.53, 64.78)	39.85 (35.14, 44.76)

^a^Numbers represent mean age and standard deviation

### Psychiatric diagnoses

Overall 41 % had no recorded diagnosis for mental health problems (MHP) prior to the suicide. Common mental disorders were recorded for 33 % of the cohort, 12 % with SMI, and 14 % with drugs and alcohol problems.

### Factors associated with a recorded diagnosis of mental illness

We examined specific predictors of having a GP-recorded mental health diagnosis (Table 2). People older than 35 years were more likely to have recorded a mental health problem, compared to their younger counterparts (OR = 4.31: 95%CI 2.37, 7.85 and OR = 5.27: CI 2.40, 11.56 for the two older age groups). People living alone or not in paid work were more likely to have a recorded mental illness. Predictably, a recorded mental health problem was more likely amongst those with a history of suicidal behaviour.

**Table 2:** association between prior diagnoses of mental health problems and predictors of suicide

	Common mental disorders	Severe mental illness	Alcohol/drugs related	All mental health diagnoses
	(*N* = 116)^a^	(*N* = 43)	(*N* = 50)	(*N* = 209)
	OR (& 95 % CI)	OR (& 95 % CI)	OR (& 95 % CI)	OR (& 95 % CI)
Gender: Male	1.00	1.00	1.00	1.00
Female	1.31 (0.61, 2.86)	0.77 (0.28, 2.17)	0.29 (0.09, 0.98)^*^	0.93 (0.45, 1.92)
Age: <35	1.00	1.00	1.00	1.00
35–54	4.37 (2.26, 8.43)^***^	2.41 (0.93, 6.27)	6.07 (2.56, 14.38)^***^	4.31 (2.37, 7.85)^***^
55+	5.93 (2.53, 13.92)^***^	4.08 (1.30, 12.86)^*^	4.63 (1.47, 14.59)^**^	5.27 (2.40, 11.56)^***^
Living alone: No	1.00	1.00	1.00	1.00
Yes	1.42 (0.75, 2.70)	2.77 (1.20, 6.39)^*^	2.91 (1.35, 6.28)^**^	1.91 (1.08, 3.40)^*^
In paid work: yes	1.00	1.00	1.00	1.00
No	2.61 (1.23, 5.56)^*^	5.78 (1.93, 17.34)^**^	4.33 (1.63, 11.51)^**^	3.27 (1.64, 6.53)^**^
Social class: professional	1.00	1.00	1.00	1.00
(Semi) routine	3.04 (0.78, 11.79)	0.30 (0.06, 1.48)	4.11 (0.46, 36.46)	2.04 (0.61, 6.14)
Other	1.58 (0.39, 6.44)	0.36 (0.07, 1.83)	1.96 (0.21, 18.76)	1.16 (0.37, 3.66)
Locale: Urban	1.00	1.00	1.00	1.00
Large towns	0.45 (0.22, 0.91)^*^	0.97 (0.37, 2.50)	0.99 (0.42, 2.32)	0.62 (0.34, 1.16)
Smaller towns	0.58 (0.19, 1.79)	1.25 (0.26, 6.10)	0.87 (0.19, 4.03)	0.72 (0.26, 1.99)
Rural	0.89 (0.42, 1.85)	1.67 (0.53, 5.22)	1.00 (0.35, 2.93)	1.00 (0.50, 1.97)
Prior attempts: no	1.00	1.00	1.00	1.00
Yes	2.21 (1.18, 4.16)^*^	1.89 (0.78, 4.59)	3.18 (1.41, 7.20)^**^	4.48 (2.35, 8.56)^***^
Consultation with psychiatrist in the twelve months prior to suicide:				
No	1.00	1.00	1.00	1.00
Yes	3.56 (1.75, 4.16)^*^	10.84 (4.22, 27.84)^***^	3.51 (1.46, 7.20)^**^	2.33 (1.32, 4.12)^**^

***: *p* = 0.000; **: *p* < 0.005; *: *p* < 0.05

^a^here N represents the number of observations included in each of the stratified analyses-the numbers of people diagnosed with CMD, SMI etc. All models fully adjusted

#### Recorded diagnosis of alcohol/drugs problems

Female suicides were less likely than males to be thus diagnosed (*p* < 0.05) while older people (55+ years) were five times more likely than young people (*p* < 0.001). Similarly, those living alone or not in paid work recorded excess likelihoods (*p* < 0.001).

#### Recorded diagnosis of severe mental illness

Middle-aged people (35–54 years) were significantly more likely to be diagnosed with SMI, compared to their younger counterparts (*p* < 0.05). Likewise, those living alone or not in paid work both recorded an excess likelihood of having a SMI diagnosis than their associated peers (*p* < 0.01).

#### Recorded diagnosis for common mental disorders

Females, people living alone, older age groups and those not in paid work were more likely than their respective counterparts to be diagnosed with an CMD (*p* < 0.05).

### Contact with general practice

Thirty-nine percent of the cohort had at least one consultation in the 30 days prior to the suicide, of whom 88 % attended for MHP. With regard to urban-rural differences we found none associated with either any type of consultation (*X*^*2*^ = 0.188, *p* = 0.98) or MHP only (*X*^*2*^ = 1.484, *p* = 0.69). This pattern is similar for the 90-day time-period in which 57 % attended a consultation, with 87 % of these attending for MHP. Finally, in the 12-months prior to suicide 82 % of the cohort consulted a GP, and of these 70 % attended for MHP. While we found no significant urban-rural differences for all consultations (*X*^*2*^ = 0.58, *p* = 0.9) there was a significant difference for MHP (*X*^*2*^ = 8.3761, *p* = 0.039).

We examined independent predictors (age, gender, living alone, paid employment, locale and social class) for GP consultations related to MHP at 1, 3, and 12 months prior to the suicide (Table 3): age (35–54 years) remained the only significant predictor of GP consultation across the three time-points. People in small towns were significantly less likely to make contact at one year only (OR = 0.18, 95%CI 0.06, 0.58). When the history of prior suicide attempts was included, age and living in a smaller town remained significant predictors at 1 year.

**Table 3:** attendance at GP services for mental health problems at one, three and twelve months before the suicide

	Attendance at GP services prior to suicide: for mental health problems			
	M1: one month prior to suicide	M2: within three months	M3:within one year prior to suicide	M4:within one year prior to suicide
	(*N* = 132)^a^	(*N* = 193)	(*N* = 293)	(*N* = 293)
	OR (& 95 % CI)	OR (& 95 % CI)	OR (& 95 % CI)	OR (& 95 % CI)
Gender: Male	1.00	1.00	1.00	1.00
Female	1.11 (0.24, 5.17)	1.13 (0.35, 3.64)	1.70 (0.79, 3.66)	1.57 (0.67, 3.64)
Age: <35	1.00	1.00	1.00	1.00
35–54	47.36 (3.58,625.8^)**^	12.92 (3.21,52.04)^***^	4.91 (2.49, 9.67)^***^	5.37 (2.57,11.24)^***^
55+	4.15 (0.81, 21.16)	4.24 (1.11, 16.13)^*^	1.76 (0.84, 3.71)	2.06 (0.91, 4.65)
Living alone:				
No	1.00	1.00	1.00	1.00
Yes	1.21 (0.30, 4.89)	2.46 (0.73, 8.27)	1.20 (0.65, 2.23)	0.93 (0.47, 1.82)
In paid work: no	1.00	1.00	1.00	1.00
yes	3.10 (0.45, 21.11)	2.70 (0.66, 10.98)	0.61 (0.30, 1.26)	0.63 (0.29, 1.34)
Social class: professional	1.00	1.00	1.00	1.00
(Semi) routine	4.84 (0.24, 97.33)	3.44 (0.65, 18.16)	1.06 (0.32, 3.51)	0.88 (0.25, 3.10)
Other	6.86 (0.30, 159.11)	3.19 (0.52, 19.39)	1.63 (0.46, 5.75)	1.41 (0.38, 5.26)
Locale:				
Urban	1.00	1.00	1.00	1.00
Large towns	1.40 (0.29, 6.74)	1.47 (0.45, 4.81)	0.86 (0.44, 1.67)	0.80 (0.39, 1.66)
Smaller towns	1.11 (0.16, 7.89)	0.70 (0.15, 3.26)	0.23 (0.08, 0.66)^*^	0.18 (0.06, 0.58)^**^
Rural	1.13 (0.19, 4.95)	0.87 (0.25, 3.01)	0.84 (0.39, 1.79)	0.99 (0.44, 2.22)
Prior attempts: no				1.00
Yes				6.76 (3.45, 13.25)^***^

***: *p* = 0.000; **: *p* < 0.005; *: *p* < 0.05

^a^Here N represents the number of observations included in each of the stratified analyses-the numbers of people attending for consultation within the specified time-period. All models fully adjusted

### Frequency of consultation

The overall mean number of consultations with GP services for any reason in the 12 months prior to the suicide was 6.74 (CI 6.45, 7.05) with males significantly less in contact (Mean = 5.92, CI 5.61, 6.25) compared to females (Mean = 9.71, CI 8.96, 10.52).

Table 4 shows the mean (or median where relevant) number of consultations for mental health problems for various risk factors associated with suicide. Of these gender, locale and prior suicide attempts recorded significantly different means within their defined categories (*P* = 0.0106, *P* = 0.0166 and *P* = 0.0195 respectively). Similarly, of the indicators with indicated non-normal distributions-social class and prior diagnosis-both recorded P-values indicating significantly different numbers of consultations within their categories.

**Table 4:** frequency of consultations for mental health problems within twelve months before suicide

	Mean or median number of GP consultations relating to mental health problems-results included for those with one or more consultation	
	Mean consultations (& standard Deviation)	F; *P*-value
Consultations	6.45 (6.88)	
Gender^a^: male	5.75 (6.05)	F = 6.66 *P* = 0.0106
Female	8.68 (8.76)	
Age^a^: <35	4.97 (4.74)	F = 0.28 *P* = 0.7587
35–54	7.06 (8.12)	
55+	7.33 (5.60)	
Living alone^a^: no	5.94 (6.00)	F = 0.67 *P* = 0.4137
Yes	7.45 (8.21)	
Paid work^a^: no	7.06 (6.31)	F = 0.19 *P* = 0.6649
yes	5.98 (7.29)	
Social class^b^:		
Professional	6.50 (7.58)	Kruskal-Wallis
Intermediate, semi-routine/routine	3.00 (6.29)	chi2 = 11.707
Other	4.00 (7.06)	*P* < 0.003
Locale type^a^: urban	5.38 (5.22)	F = 2.81 *P* = 0.0166
Larger town	8.42 (9.28)	
Small towns & rural - combined	5.52 (4.70)	
Prior suicide attempts^a^: no	6.16 (7.43)	F = 3.38 *P* = 0.0195
Yes	6.64 (6.52)	
Prior diagnosis^b^: none	2 (4.69)	Kruskal-Wallis
Common metal disorders	4.5 (6.71)	chi2 = 16.818
Serious mental illness	3 (7.34)	*P* < 0.001
Alcohol/drugs	6 (8.30)	

^a^Variable normally distributed in cohort - means (& Standard Deviation) used to describe number of consultations

^b^ Variable not normally distributed in the cohort - median used to describe the number of consultations

### GP concern for patient suicidality

We examined the GP records for any indication that the GP was concerned about a patient’s possible suicidality. Table 5 shows the results of a series of logistic regression models to examine for predictors of GP concern of suicidality, excluding those patients who had recorded a prior suicide attempt. In the univariate analyses those most likely to raise GP concern included: patients aged 35–54 years (OR = 3.31: 95%CI 1.61, 6.79); patients with a prior diagnosed mental illness-especially alcohol/drugs problems and serious mental illness (OR = 10.15: 3.45, 29.88 and OR = 11.08: 3.39, 36.20 respectively); and males, approximately twice as likely as females. In the main models (M1 to M4) recording incremental adjustment for socio-demographic and clinical factors, frequency of consultations remains a dominant predictor of GP concern for suicidality (OR = 1.18: 1.07, 1.31) in all the models. Similarly, GPs were about five times less likely to document concern for female suicidality (when compared to males) (OR = 0.18, 0.04, 0.81). Finally, a prior MH diagnosis remains highly predictive in all models.

**Table 5:** Factors related to general practitioner vigilance to suicidality^a^

	Univariate analysis^b^	M1: adjusted for consultations, age & gender	M2: M1 + diagnosis	M3: M2 + socio-demographic	M4: M3 + locale
GPconsultations: number^c^	1.18 (1.10, 1.27)^***^	1.24 (1.12, 1.36)^***^	1.20 (1.08, 1.33)^***^	1.20 (1.08, 1.33)^**^	1.18 (1.07, 1.31)^**^
Age: 35–54 (ref = < 35)	3.31 (1.61, 6.79)^***^	2.09 (0.94, 4.62)	1.25 (0.51, 3.03)	1.20 (0.48, 2.99)	1.20 (0.47, 3.11)
55+	2.11 (0.86, 5.20)	0.67 (0.21, 2.18)	0.50 (0.14, 1.79)	0.49 (0.13, 1.88)	0.52 (0.13, 2.15)
Gender: female (ref = male)	0.50 (0.18, 1.38)	0.18 (0.04, 0.72)^*^	0.19 (0.05, 0.79)^*^	0.21 (0.05, 0.89)^*^	0.18 (0.04, 0.81)^*^
MH problems: (ref = none)					
Common MH problems	5.24 (2.36, 11.62)^***^		2.75 (1.06, 7.14^)*^	2.86 (1.09, 7.53^)*^	3.47 (1.25, 9.66)^*^
Serious mental illness	11.08 (3.39, 36.20)^***^		7.76 (2.03, 29.67)^**^	8.61 (2.13, 34.85)^**^	8.08 (1.94, 33.71)^**^
Drugs/alcohol problems	10.15 (3.45, 29.88)^***^		4.34 (1.18, 16.0)^*^	4.74 (1.23, 18.17)^*^	4.28 (1.13, 16.25)^*^
Lives alone: yes (ref = no)	1.35 (0.67, 2.68)			0.85 (0.34, 2.12)	0.74 (0.28, 1.95)
In paid work: no (ref = yes)	0.87 (0.45, 1.65)			0.79 (0.34, 1.87)	0.90 (0.37, 2.22)
Locale: large towns (ref = urban)	1.99 (0.94, 4.22)				2.35 (0.93, 5.60)
Small towns^d^	na				na
Rural areas	1.65 (0.74, 3.65)				1.65 (0.60, 4.56)

*** = *p* < 0.001; ** = *S* < 0.005; * = 0.05

^a^ Analysis includes only those who have no history of prior attempts

^b^ Data represents Odds Ratios (ORs) indicating the likelihood of the patient having discussed suicide with their GP

^c^ Number of consultations entered into the model as continuous-ORs represent the increase in likelihood of engagement per additional consultation

^d^ The category ‘small towns’ predicts the outcome perfectly-they are therefore dropped from the analysis

### Contact with other services

While 33 % of the cohort were in contact with nursing services, only 7 % of these related to MHP; 17 % had contact with social workers, of which 92 % were mental health-related; and 39 % had contact with Accident and Emergency services, of whom 58 % had recorded a mental health problem. Just over a third had seen a psychiatrist in the 12 months prior to suicide; and 28 % had been in contact with community mental health services in the prior 12 months. We found no differences across type of services for urban–rural locales. Only 2 % of the cohort were recorded as having received counselling services.

## Discussion

Consistent with other evidence, we found that a majority of people who die by suicide have been in contact with primary care within 12 months prior to death. This study offers an enriched understanding of GP contact in that we were able to ascertain the nature of these consultations and their frequency. The majority of contacts were related to mental health problems, with this increasing over time. The likelihood of contact is increased among women, older people and among patients identified as having substance misuse problems [6] and common mental disorders. It is worth noting that people with Severe Mental Illness were less likely to be seen by GPs at any time over the 12 month period than those with CMD and substance misuse problems. Although overall, while women were significantly more likely to be in contact with primary care for any reason, we found no gender differences specifically in relation to mental health contacts.

Despite the high level of contact for mental health related problems, we found that GP recorded mental health diagnoses (40 %) were similar to that described elsewhere (NCISH, 2014). This may be explained by either GP underestimation of the severity of problems and/or an unwillingness to formally diagnose. While many patients conceal or downplay the severity of symptoms, the detection of suicidal intent remains a major challenge. This is not just a problem within primary care. Thus, while many people had been in contact with a nurse, this predominantly concerned non-mental health problems. When considering the suicidality of patients GPs tended to be strongly influenced by the gender of the patient and previous psychiatric history. While this may appear a legitimate response - acknowledgement of well-known risk factors - it appears to be based on a degree of profiling which may be at the expense of patients without these characteristics.

Worryingly, only 2 % of patients appeared to be in receipt of counseling services. However, it is likely that other people will have sought such services privately or were referred to third sector agencies. Unfortunately, primary care appears not to have any connection with, or feedback from, these external services.

Recent evidence from various national settings suggests a general trend of increasing rural suicide when compared to urban areas [10–13]. The evidence for differences in urban-rural suicide mortality in Northern Ireland is less clear [14, 15]. Our analysis of urban-rural locale was based on a graded four-fold classification, allowing for more nuanced urban-rural analysis. We found that the highest suicide rates were recorded for those in larger towns. Explanations for this excess are lacking: there is no evidence that socio-economic problems are worse in towns relative to other locales or that service provision is poorer [16]. In addition to the higher suicide rates within towns,we found a significantly higher level of primary care contact among all suicides, particularly among women. It may be that towns have a relatively underdeveloped mental health provision within the voluntary and private sectors, compared to cities, placing a greater demand on primary care services.

## Conclusions

These findings suggest that too high a proportion of suicidal people remain undiagnosed and untreated for mental health problems. Every contact with professional health services represents a potential opportunity for recognising suicidality among patients. A minority of people who take their own lives appear not to make contact with medical professionals of any type and apparently complete the suicide at the first attempt [17]. Nevertheless these findings suggest that contact with services among a majority of suicides may be considerable and, consonant with other research, we suggest that frequent attendance can be a marker for risk, as can receiving different kinds of medication for mental health problems [6]. However, suicide remains a rare event and screening for suicide among high-risk patients may not be a cost-effective suicide prevention strategy [18, 19].

### Limitations

A limitation of studies such as this is that we lack an appropriate comparison group. Although we cannot say whether this population differed substantially in their contact with services than the general population, they appear to have visited their GP much more often [5]. Although we were unable to obtain GP records for 9.5 % of the suicides, these are unlikely to have significantly affected the overall findings. As noted previously, rural environments vary considerably ensuring that international comparisons are problematic. In any case, we are unable to address the ‘life-history’ transition of people from one locale to another. The lack of standardisation in GP terminology for drug and alcohol problems presents a challenge to studies of this kind and, therefore, we were unable to distinguish any primary concern of the GP, if one existed. Moreover, the diagnostic data is derived from the International Classification of Primary Care (ICPC-2, which is limited to a primary diagnosis only. Co-morbidity may be an additional risk factor in determining help seeking-perhaps more so for those with undetected (and therefore, undiagnosed drug or alcohol problems).

Lastly, while our study benefits from the additional more finely grained data from GP records, rather than those from coroner’s records only, missing data on contact with other services remains a possibility.

### Ethics

This study was granted a favourable opinion by the Office for Research Ethnics Northern Ireland (ORECNI), a branch of the National Research Ethics Service in the United Kingdom (REC reference: 10/NIR03/65).

### Consent to publish

Permission to access the required data from Coroners Service Northern Ireland and General Practice records was granted under Section 33 of the Data Protection Act.

### Availability of data and materials

The data can be made available by agreement with the principal investigator.

## Abbreviations

CMD: common mental disorders
CSNI: Coroner Service for Northern Ireland
GP: general practitioner
ICPC-2: International Classification of Primary Case (ICPC-2)
MHP: mental health problems
NISRA: Northern Ireland Statistics and Research Agency
PTSD: posttraumatic stress disorder
UK-ORECNI: United Kingdom Office of research ethic committees (Northern Ireland)
